# Experimental Models for the Study of Central Nervous System Infection by SARS-CoV-2

**DOI:** 10.3389/fimmu.2020.02163

**Published:** 2020-08-28

**Authors:** Inmaculada Sanclemente-Alaman, Lidia Moreno-Jiménez, María Soledad Benito-Martín, Alejandro Canales-Aguirre, Jordi A. Matías-Guiu, Jorge Matías-Guiu, Ulises Gómez-Pinedo

**Affiliations:** ^1^Laboratory of Neurobiology, Department of Neurology, Institute of Neurosciences, San Carlos Institute for Health Research, Universidad Complutense de Madrid, Madrid, Spain; ^2^Preclinical Evaluation Unit, Medical and Pharmaceutical Biotechnology, CIATEJ-CONACYT, Guadalajara, Mexico

**Keywords:** SARS-CoV-2, experimental models, COVID-19, central nervous system, neurodegenerative disease, multiple sclerosis

## Abstract

**Introduction:**

The response to the SARS-CoV-2 coronavirus epidemic requires increased research efforts to expand our knowledge of the disease. Questions related to infection rates and mechanisms, the possibility of reinfection, and potential therapeutic approaches require us not only to use the experimental models previously employed for the SARS-CoV and MERS-CoV coronaviruses but also to generate new models to respond to urgent questions.

**Development:**

We reviewed the different experimental models used in the study of central nervous system (CNS) involvement in COVID-19 both in different cell lines that have enabled identification of the virus’ action mechanisms and in animal models (mice, rats, hamsters, ferrets, and primates) inoculated with the virus. Specifically, we reviewed models used to assess the presence and effects of SARS-CoV-2 on the CNS, including neural cell lines, animal models such as mouse hepatitis virus CoV (especially the 59 strain), and the use of brain organoids.

**Conclusion:**

Given the clear need to increase our understanding of SARS-CoV-2, as well as its potential effects on the CNS, we must endeavor to obtain new information with cellular or animal models, with an appropriate resemblance between models and human patients.

## Introduction

On 31 December 2019, the Word Health Organization reported for the first time on an epidemic of lower respiratory tract infection in Wuhan, in the Chinese province of Hubei. The causal agent was soon identified as Severe acute respiratory syndrome coronavirus (SARS-CoV-2), a coronavirus (CoV), and the associated disease was named coronavirus disease 19 (COVID-19) (1). CoVs are positive-sense single-stranded RNA viruses resembling a crown under microscopy due to the presence of spike (S) glycoproteins on the viral envelope. There are four types of CoVs: αCoVs, βCoVs, δCoVs, and γCoVs. SARS-CoV, MERS-CoV, and SARS-CoV-2 are zoonotic (2), first infecting animals and then spreading to humans. βCoVs, which include SARS-CoV-2, are thought to originate in bats (3, 4), among other species. These viruses can cause respiratory and enteric diseases in different animal species. In humans, HCoV-OC43 and HCoV-HKU1 (αCoVs), and HCoV-229E and HCoV-NL63 (βCoVs) can cause the common cold and self-limiting infection of the lower respiratory tract in immunocompetent individuals in seasonal periods (5). Two previous epidemics have, however, been caused by CoVs; these were similar to the current pandemic, though with higher mortality rates, and were caused by the severe acute respiratory syndrome coronavirus (SARS-CoV) and middle east respiratory syndrome coronavirus (MERS-CoV) and affected many countries (6). Although they led to many research studies, expanding our knowledge of the viruses, the COVID-19 epidemic has had a far greater global impact. One as-yet unknown aspect of SARS-CoV-2 is its potential short- and long-term impact on the central nervous system. In this respect, although the rate of neurological symptoms associated with infection is not high (7), the possibility of subsequent effects of central nerve system (CNS) infection has been suggested by many authors (8–17). To address these questions, we need experimental models based on advances in biomedical research both *in vitro* and *in vivo*. We review these models in the present article.

## Objectives of the Experimental Models in the Study of the Impact of SARS-CoV-2 Infection

Previous studies on SARS-CoV already demonstrated that the spike protein facilitates viral invasion of the target cell by interacting with the angiotensin converting enzyme 2 (ACE2) (18). This protein is expressed on the surface of epithelial cells of the lower respiratory tract, endothelial cells of arteries and veins, intestinal mucosa cells, kidney cells, immune cells, glial cells, and neurons (19–21). *In vitro* studies have demonstrated that SARS-CoV-2 also uses this receptor to penetrate cells (22, 23). In fact, sequencing of the SARS-CoV-2 spike protein has shown that it contains residues that may increase its binding affinity to the ACE2 receptor 10- to 20-fold in comparison with SARS-CoV (18). When spike protein interacts with the receptor, cleavage and activation by cell proteases is necessary to enable the viral membrane to fuse with the host cell and the virus to penetrate. *In vitro* studies have shown that SARS-CoV-2 spike proteins are mainly processed by transmembrane protease serine 2 (*TMPRSS2*), by endosomal cathepsins B and L in the absence of *TMPRSS2* (23, 24), and by furin (25, 26). Once the virus is internalized, viral RNA is released into the cytoplasm, and a series of translations and replications create new RNAs and viral proteins that assemble to form new virions (20). These are released from the host cell and can infect new target cells expressing the appropriate receptor on their surfaces (20, 23).

More complex biological systems are, however, needed to study the possible interactions between SARS-CoV-2 and the host. Due to these complex interactions, selecting an appropriate model should be a thoughtful and clearly defined process, in order to provide relevant and translatable scientific data enabling us to address the questions of interest and to ensure the rational use of animals. Although many animals may respond similarly to humans from physiological, pathological, and therapeutic perspectives, we must bear in mind that differences between species may lead to erroneous conclusions (27); it is therefore necessary to understand the relationship between the model and the human disease (28). In the light of the current pandemic, it is essential to have a cellular or animal model mimicking the symptoms and pathological processes identified in patients infected with SARS-CoV-2 (29).

## *In Vitro* Models (Table 1)

### Vero E6 Cell Line

This cell line was isolated from kidney epithelial cells extracted from an African green monkey (*Chlorocebus aethiops*) in 1979; the Vero E6 cell line has been shown to be very useful for viral propagation and production *in vitro* (30). These cells are permissive to SARS-CoV replication, as they efficiently express the ACE2 receptor (31, 32). Furthermore, they enable persistent infection *in vitro* (33). Vero E6 cells have therefore already been used in studies with SARS-CoV and MERS-CoV (30, 33–36) and also in the development of live-attenuated and inactivated vaccines for human use (37, 38). More recently, they have been used in research into the viral infection mechanism in COVID-19 (23, 39, 40), the effects of the virus on cells, confirmation of viral infection (41–43) and pharmacological research (44–48).

**TABLE 1:** *In vitro* models for the study of SARS-CoV-2.

**References**	**Cell Line**	**Origin**	**Virus Dose**	**Use**
(23, 42, 43, 44, 47, 48)	***Vero E6***	African green monkey (*Chlorocebus aethiops*) kidney epithelial cells	•**Multiplicity of infection (MOI) of 0.01 to 0.5**	•*In vitro* amplification of viral particles •Diagnosis of SARS-CoV-2 infection and viral isolation •Study of infection mechanism •Search for new therapeutic targets •Pharmacological screening
(23, 24, 39, 40)	***HEK 293T***	Human embryonic kidney epithelial cells	•**No determined**	•Study of infection mechanism •Search for new therapeutic targets •Pharmacological screening
(23, 39)	***BHK-21***	Baby hamster (*Mesocricetus auratus*) kidney fibroblasts	•**8 × 10^7^ genome equivalents (GE) per 24-well of SARS-CoV-2**	•Study of infection mechanism
(23, 26, 46, 48)	***Huh7***	Human hepatocellular carcinoma	•**MOI of 0.05 to 1**	•Diagnosis of SARS-CoV-2 infection and viral isolation •Study of infection mechanism •Pharmacological screening
(39, 41, 43)	***LLC-MK2***	Rhesus macaque (*Macaca mulatta*) kidney epithelial cells	•**No determined**	•Diagnosis of SARS-CoV-2 infection and viral isolation •Study of infection mechanism
(23)	***Caco-2***	Human colorectal adenocarcinoma	•**No determined**	•Study of infection mechanism
(23, 39, 72)	***Calu-3***	Human lung adenocarcinoma	•**MOI of 0.001 to 0.08**	•Study of infection mechanism •Pharmacological screening
(116, 117, 139, 141)	***Human brain organoids***	Human induced pluripotent stem cells (hIPSCs)	•**MOI of 0.1 to 2.5**	•Nervous tissue infection mechanisms •Neurodegenerative mechanisms •Pharmacological screening

*The table includes the main cell lines used in the study of SARS-CoV-2 infection, the origin of the cells, viral dose used in some studies for the infection of cells and their uses. The culture media used for each cell line is specified in Supplementary Material.*

### HEK 293T Cell Line

This cell line is a variant of the HEK 293 lineage, which was isolated from kidney epithelial cells extracted from human embryos (49). Both lines are widely used in research due to their high transfectability, gene expression, and production of proteins or recombinant retrovirus (23, 49, 50). The HEK 293T variant expresses the T SV-40 antigen, which enables the amplification of transfected plasmids containing the SV40 origin of replication and thus considerably increases the expression levels of desired gene products (50). These cells have also previously been used in studies of other such viruses as herpes simplex virus, SARS-CoV, and even CoV pseudovirions (34, 51–53). Due to its high efficiency of transfection, research with this cell line is producing significant findings, such as the confirmation of ACE2 as the cell entry receptor for SARS-CoV-2 and the potential role of CD147 as an alternative receptor (23, 24, 39, 40).

### BHK-21 Cell Line

This line is a subclone of the fibroblast cell line extracted from 1-day-old Syrian hamster (*Mesocricetus auratus*) kidney cells (54) and is useful for studying virus propagation and plasmid transfection (55). It has been used in the study of HCoV-OC43 and SARS-CoV-2 infection mechanisms (23, 36, 56, 57).

### Huh7 Cell Line

This lineage was established in 1982 from a human hepatocellular carcinoma; it is therefore able to produce a great variety of substances secreted by the human liver (58) and has been used in the study of hepatitis C virus, SARS-CoV, and MERS-CoV (27, 34, 58, 59). In the context of SARS-CoV-2, this cell line has been used in the study of infection mechanisms (23, 39), the cytotoxicity of viruses from human hosts (22, 60), and in pharmacological research (26, 46, 48).

### LLC-MK2 Cell Line

The LLC-MK2 cell line was established in 1955 from rhesus macaque (*Macaca mulatta*) kidney epithelial cells (61). It has been mainly used in the study of poliovirus, but also to study SARS-CoV, HCoV-NL63 (34, 62, 63), and SARS-CoV-2 to assess the cytopathic effects in patients infected with the virus (41, 43) and to characterize its viral infectious pathway (39).

### Caco-2 Cell Line

This line was extracted in 1977 from human epithelial colorectal adenocarcinoma cells; under specific culture conditions, the cells are able to differentiate into small intestine enterocytes (64). This cell line is frequently used to study transfection, invasion, and absorption (65). It has been used in studies of MERS-CoV, SARS-CoV, and HCoV-NL63 (27, 66, 67), and in the analysis of the S protein activation in studies with SARS-CoV-2, due to the fact that these cells express the TMPRSS2 protease (23).

### Calu-3 Cell Line

This line was extracted in 1975 from a human lung adenocarcinoma and is used in the study of respiratory viruses (68, 69). It has been used in the study of SARS-CoV and MERS-CoV (27, 70, 71) and infection mechanisms studies and pharmacological research into COVID-19 (23, 39, 72).

### Other Cell Lines

Several of the available cell lines susceptible to transfection may also be used in *in vitro* research. For example, the study by Zhou et al. (22) uses the *HeLa* cell line, which does not endogenously express ACE2, and expression plasmids for human ACE2 (hACE2) in the study of SARS-CoV-2.

## *In Vivo* Models (Table 2)

Mouse and hamster strains are the main animal models used in research into SARS-CoV and MERS-CoV (73); however, in the case of SARS-CoV-2, research efforts have been focused on the study of aged mouse strains, the design of humanized mouse models that express the hACE2 receptor, and the creation of knockout mice, with the aim of replicating the mechanisms involved in human infection.

**TABLE 2:** *In vivo* models for the study of SARS-CoV-2 infection in the CNS.

**References**	**Model**	**Virus, inoculation route, and dose**	**Findings**
(76)	C57BL/6 *ACE2* knockout mouse	SARS-CoV; intranasal; 7.6 × 10^6^ pfu/ml in DMEM	Mechanisms of primary infection and viral propagation
(73)	C57BL/6 *TMPRSS2*–/– knockout mouse	SARS-CoV; intranasal; 10^5^ TCID_50_ in F-musX MERS-CoV; intranasal; 10^6^ TCID_50_ in HCoV-EMC 2012	Mechanisms of primary infection and viral propagation in the respiratory tracts
(83, 85)	C57BL/6 *STAT1* knockout mouse	SARS-CoV, SARS-CoV 2; intranasal 7 × 10^4^ pfu	Cytokine-induced biological responses
(79)	hACE2 ICR mouse	SARS-CoV-2; intranasal; 10^5^ TCID_50_	Viral antigen in the lower respiratory tract, alveolar infiltrates, and weight loss
(80)	C57BL/6J-hACE2-AAV mouse	SARS-CoV-2; intranasal; 3 × 10^7^ pfu/ml	Pulmonary infiltrate, neutralizing antibodies, interferon-stimulated genes
(81)	C57BL/6 young and aged hACE2 mouse	SARS-CoV-2; intranasal; 4 × 10^5^ pfu. Intragastric; 4 × 10^6^ pfu	Viral antigen in trachea, lungs, and brain. Pulmonary infiltrate. Pathological changes in lungs are more evident in aged mice.
(88)	Young and aged BALB/c mouse	Adapted viruses: SARS-CoV-2 MA; intranasal; 10^5^ pfu	Viral replication in lower and upper respiratory tract. Clinical symptoms worsen with age. Prophylactic and therapeutic role of interferon lambda-1a
(87)	Young and aged BALB/cC57 mouse Young C57BL/6 mouse	Adapted viruses: SARS-CoV-2 MASCp6; intranasal; 7.2 × 10^5^ pfu Adapted viruses: SARS-CoV-2 MASCp6; intranasal; 7.2 × 10^5^ pfu	Evaluation of the efficacy of the subunit vaccine consisting of SARS-CoV-2 S protein RBD fused with a human IgG Fc subunit
(92)	Syrian hamster (*Mesocricetus auratus*)	SARS-CoV-2; intranasal; 8 × 10^4^ TCID_50_	Viral antigen in the upper and lower respiratory tracts and intestinal mucosa. Pulmonary infiltrates. Neutralizing antibodies. Transmission by direct contact or aerosols
(146, 148)	C57BL/6 mouse	MHV coronavirus; intranasal, intracerebral, enteral (1–2.5 × 10^3^pfu)	Mechanisms of primary infection and viral propagation, demyelinating lesion model
(93)	Syrian hamster (*Mesocricetus auratus*)	SARS-CoV-2; intranasal; 10^5^ pfu in DMEM	Viral antigen in the upper and lower respiratory tracts and intestinal mucosa. Mononuclear infiltrates. Neutralizing antibodies. Transmission by direct contact
(97)	*Mustela putorius furo*	SARS-CoV-2 F13-E and SARS-CoV-2 CTan-H; intranasal; 10^5^pfu	Viral replication in the upper respiratory tract without causing severe disease
(98)	*Mustela putorius furo*	SARS-CoV-2 GISAID ID EPI_ISL 406862; intranasal; 6 × 10^5^ TCID_50_ of SARS-CoV2 virus diluted in 500 μl of PBS	Direct and indirect transmission
(96)	*Mustela putorius furo*	SARS-CoV strain Toronto-2; intranasal; 10^3^TCID_50_/mL diluted in medium	Transmission before peak viral load, without symptom onset
(105)	*Macaca fascicularis*	SARS-CoV-2 # 026V-03883, MERS-CoV # 011V-02838; intratracheal, intranasal; 10e^6^ TCID_50_ in PBS	Clinical symptoms similar to COVID-19
(100)	*Macaca mulatta*	SARS-CoV-2 HB-01; intratracheal; 10^6^ TCID_50_/mL	More severe clinical symptoms in older monkeys
(106)	*Macaca mulatta*	SARS-CoV-2 MN985325.1; intratracheal, intranasal, conjunctival, oral; 4 × 10^5^ TCID_50_/ml of DMEM	Significant increase in pro-inflammatory interleukins
(109)	*Macaca mulatta*	SARS-CoV-2 WH-09/human/2020/CHN; conjunctival; 1 × 10^6^ TCID_50_/ml	Viral load in the ocular system and respiratory and digestive tracts
(107)	*Macaca mulatta/Macaca fascicularis/Callithrix jacchus*	SARS-CoV-2; intratracheal, intranasal, conjunctival; 10^6^ pfu/ml	Rhesus monkey is more susceptible to infection.
(110)	*Macaca mulatta*	MERS-CoV strain HCoV-EMC/2012; intratracheal; 7 × 10^6^ TCID_50_	Viral RNA in the throat soon after infection
(111)	*Macaca mulatta*	SARS-CoV-2/WH-09/human/2020/CHN; intratracheal; 1 × 10^6^ TCID_50_/ml	Rhesus monkeys present no reinfection
(112)	*Macaca mulatta*	SARS-CoV-2 NR-52281; intratracheal, intranasal; 1.1 × 10^6^ pfu or 1.1 × 10^5^ pfu or 1.1 × 10^4^ pfu	No reinfection
(113)	*Macaca mulatta*	SARS-CoV-2 MN985325.1; intratracheal, intranasal, conjunctival, oral; 2.6 × 10^6^ TCID^50^	Treatment study
(115)	*Macaca mulatta*	SARS-CoV-2; intratracheal; 10^6^ TCID_50_/ml	Vaccine development

*The table includes the characteristics of the animals (species) used, as well as the route of administration, viral dose used in some studies for the infection and the relevance of the model in SARS-CoV-2 research. *TCID50*, 50% tissue culture infectious dose; *pfu*, plaque forming units.*

### Aged Mouse Strains

Roberts et al. (74) reported that aged BALB/c mouse strains were able to maintain high rates of viral replication, which was associated with clinical illness and pneumonia; the study demonstrated an age-related susceptibility to SARS disease in animals that parallels the human experience. Advanced age has been identified as an independent factor of poor prognosis in COVID-19 and is considered a predictor of mortality in patients with SARS-CoV-2 infection. Other strains like C57BL/6 have also been used (75).

### Knockout Mouse Models

K18-hACE2 transgenic mice express hACE2, with regulation by the human cytokeratin 18 (K18) promoter. Specifically, these mice contain 2.5 kb of the K18 genomic sequence, including the promoter, the first intron, and a translation-promoting sequence. Expression of hACE2 is mainly observed in the epithelium of the respiratory tract, which shows a higher incidence of SARS-CoV-2 infection, and in the epithelium of other organs, including the liver, kidneys, and gastrointestinal tract (76). K18-hACE2 mice infected with SARS-CoV-2 show weight loss and viral replication in the lungs, as in humans. Furthermore, they show the typical histopathological findings of interstitial pneumonia with lymphocytic and monocytic infiltration into the alveolar interstitium and an accumulation of macrophages in the alveolar cavities. Alveolar and bronchial epithelial cells show presence of viral antigens; this is not observed in wild-type (WT) mice with SARS-CoV-2 infection. Furthermore, these mice show other pathological changes, such as vasculitis, degeneration, and necrosis of extrapulmonary organs and presence of the viral antigen in the brain (77, 78). Bao et al. (79) recently studied the pathogenicity of SARS-CoV-2 virus in hACE2-expressing ICR transgenic mice and WT mice. This model may be useful in research into drug treatments for COVID-19. Israelow et al. (80) developed an hACE2-adeno-associated virus 9 (AAV) murine model, which was subsequently intranasally infected with SARS-CoV-2. They studied the presence of coronavirus infection, the inflammatory response in the lungs, the presence of neutralizing antibodies, and the type I interferon signaling pathway. The authors reported that this model would be very useful for understanding questions related to infection, replication, and pathogenesis of SARS-CoV-2 and for testing therapeutic strategies.

Sun et al. (81) used *CRISPR/Cas9* technology to create a murine hACE2 model in young and aged animals. After intranasal infection of the animals with SARS-CoV-2, the researchers observed viral replication in the lungs, trachea, and brain of both animals. However, alveolar inflammatory infiltrate and vascular lesions were more evident in aged animals; this is analogous to the pathological changes observed in older patients with COVID-19. This model also showed evidence of respiratory tract infection following intragastric inoculation.

### *TMPRSS2*–/– Knockout Mice

This model was designed using a directional vector to replace exons 10–13, which codify the serine protease domain of the *TMPRSS2* gene. It was constructed by electroporation of embryonic stem cells and their subsequent injection into C57BL/6 blastocysts for at least five generations (82). After experimental infection with SARS-CoV-2, *TMPRSS2*-deficient mouse strains showed reduced body weight loss and viral kinetics in the lungs. Absence of *TMPRSS2* affected the infection sites and virus spread within the respiratory tract; therefore, this is a useful model for COVID-19 research (73).

### The *STAT1* Knockout Mouse Model

(129S6/SvEv-STAT1tmRDS) contains a homozygous *STAT1* mutation and completely lacks functional *STAT1* proteins (Pgm1c and Gpi1b alleles of 129S6). The model was created by targeting the *STAT1* gene in GS-1 ES cells and injecting target cells into blastocysts. Heterozygous models of the mutation were produced from the chimeras and were crossed over to generate homozygous models (83, 84). The *JAK-STAT* signaling pathway is involved in the mediation of cytokine-induced biological responses. This is therefore a useful model for determining the role of a variety of cytokines in immune responses, the role of *STAT1* protein in mediating interferon-dependent responses, and its relationship with viral and bacterial pathogens (84–86); the model is also interesting in the analysis of SARS-CoV-2 inflammation mechanisms.

### Adapted Mouse Models

Studies have been recently conducted with BALB/c and C57BL/6 mice (87, 88) together with modified SARS-CoV-2 strains. Modification of the virus has led to mouse-adapted SARS-CoV-2 strains, the SARS-CoV-2 MA (88) and SARS-CoV-2 MASCp6 (87) strains, which are able to infect mice with no need for modification of the animals, as these strains efficiently bind to the murine ACE2 receptor in both young and aged mice, causing a disease resembling human COVID-19. In addition to their use in the study of pathogenesis, these models have enabled researchers to trial vaccines and treatments for the disease (87, 88).

### Mouse Models for the Induction of Neutralizing Antibodies

BALB/c mice have been used in studies for the development of vaccines, and Wistar rats have been used in studies into immunization with attenuated strains of the virus (89).

### Syrian Hamster Models

This animal model (*M. auratus*) has previously been used in the study of SARS-CoV (90, 91), as the hamster presents an ACE2 receptor homologous to the human receptor. Sia et al. (92) intranasally inoculated animals with the β–CoV/Hong Kong/VM20001061/2020 strain and corroborated the presence of viral antigen in the epithelial cells of the nasal and bronchial mucosa with progression to pneumocytes and clearance of infectious particles by day 7 after infection. Presence of mononuclear cell infiltrates was moderate in the nasal turbinates but was greater in the lungs. The viral antigen was observed in epithelial cells of the duodenum, without signs of inflammation. No infectious particles were detected in the kidney, and no histopathological changes were observed in other organs. Neutralizing antibodies were observed on day 14 after infection. Infected animals presented clear weight loss and researchers corroborated that the main route of viral transmission was through direct contact or aerosols. The authors conclude that SARS-CoV-2 infection in Syrian hamsters presents similar characteristics to those observed in humans with mild infections and that this model represents an opportunity for understanding the transmission dynamics of this novel coronavirus.

Chan et al. (93) confirmed transmission by contact, the progressive decrease in viral load between days 2 and 7 after infection in both lower and upper respiratory tracts, and the expression of viral antigens (protein N) by epithelial cells together with presence of mononuclear infiltrates. On day 7 after infection, regenerative hyperplasia occurring in bronchioles led to the appearance of multiple irregularly arranged epithelial layers. Furthermore, they detected the viral antigen in the intestinal mucosa and observed histopathological changes in the spleen and heart. The pro-inflammatory cytokine cascade in this model normalizes at approximately day 7, and antibodies are observed at 7–14 days. This animal model reproduces the respiratory and enteric symptoms observed in patients with COVID-19.

### Ferret Models

Ferrets (*Mustela putorius furo*) are frequently used as animal models to study respiratory diseases caused by such viruses as influenza virus or SARS-CoV (94–96). All studies with SARS-CoV-2 in ferrets have used the intranasal route to inoculate the virus (97–99). Shi et al. (97) studied the susceptibility of ferrets to infection with 2 SARS-CoV-2 strains. In a first stage of the study, the authors detected viral RNA in samples from the nasal turbinates, palate, and tonsils, but no viral load was detected in the other organs analyzed, including the lungs and brain. This suggests that the virus can replicate in the upper respiratory tract of ferrets. In a second stage of the study, analyzing the viral replication dynamics, animals showed viral RNA in nasal washes, whereas rectal swabs showed much lower viral load. Furthermore, ferrets presented anti-SARS-CoV-2 antibodies at days 13 and 20. Histopathological studies showed altered pneumocytes, macrophages, and neutrophils. Finally, when studying replication in the lungs, the authors observed that the virus is able to replicate in the upper respiratory tract up to day 8 after infection without causing severe symptoms. Richard et al. (98) focused on the study of the direct and indirect transmission of the SARS-CoV-2. Ferrets inoculated with SARS-CoV-2 were placed in direct contact with a group of healthy ferrets 6 h after infection. Inoculated animals showed productive infection, and the peak of viral infection was reached on the third day. All animals in direct or indirect contact presented viral RNA at day 1–3 after exposure. Animals exposed to inoculated ferrets were expected to present a lower viral load than the inoculated animals; however, viral load was similar in all cases. Lastly, the authors observed that all animals presented antibodies at day 21 after exposure to the virus, and that their levels were similar, regardless of the form of infection. A similar design was employed by Kim et al. (99), who observed viral load up to 8 days after infection, both in samples from inoculated animals and in samples from animals that were in direct contact with the inoculated ferrets. Animals in indirect contact with infected ferrets showed positive results for infection at day 2 after exposure, which suggests rapid transmission, even before peak viral load was reached and in the absence of clinical symptoms; this correlates with the reported transmission by asymptomatic human patients.

### Non-human Primate Models

Severe acute respiratory syndrome coronavirus and middle east respiratory syndrome coronavirus have previously been studied in non-human primates (75, 100–104); the use of these models has been proposed for the study of SARS-CoV-2 (105) especially with a view to the development of vaccines or antiviral treatments (106, 107). Rockx et al. (105) used a combined intratracheal and intranasal route to inoculate the SARS-CoV-2 virus to both young and old adult cynomolgus macaques (*Macaca fascicularis*). The results of this study showed that these animals tolerated viral infection, presenting symptoms similar to those of COVID-19 in humans. The virus efficiently replicates in epithelial cells throughout the upper respiratory tract, which correlates with the ease of transmission of the virus, whereas replication in the lower respiratory tract correlates with the development of the disease (105–108). Yu et al. (100) obtained similar results with intratracheal inoculation of young and old rhesus monkeys (*Macaca mulatta*). The authors report that, although viral replication was more active in older adult monkeys, both groups developed interstitial pneumonia together with edema, which was more severe and diffuse in older adult monkeys. Munster et al. (105) developed a COVID-19 model using rhesus monkeys, inoculating them with the virus through four different but combined routes (intranasal, intratracheal, conjunctival, and oral). The animals presented clinical signs compatible with COVID-19 from day 1 after the inoculation to symptom resolution, between days 9 and 17. All animals presented weight loss, low-grade fever, and pulmonary infiltrates, in addition to a significant increase in IL-6 and IL-10, among other findings; IgG antibodies were present at detectable levels from day 7 after the infection. Nasal, pharyngeal, and rectal samples showed high levels of viral RNA. Furthermore, histopathological analyses showed similar alterations to those caused by SARS-CoV and MERS-CoV (97, 98). Deng et al. (109) inoculated rhesus monkeys with SARS-CoV-2 through the conjunctival route. They did not observe significant changes in the animals’ weight or body temperature. Antibody analyses detected presence of IgG at days 14 and 21. Furthermore, radiographs of these animals showed bilateral alterations in the upper lobes and right lower lobe, which correlates with the moderate interstitial pneumonia observed microscopically. Histopathological studies showed viral load in the ocular and nasolacrimal system, as well as in the respiratory and digestive tracts. Results of a comparative study of three non-human primate species [rhesus monkey, crab-eating macaque, and common marmoset (*Callithrix jacchus*)], which were inoculated with the virus through three different pathways (intratracheal, intranasal, and conjunctival), showed that almost all animals presented clinical signs compatible with COVID-19, although there were differences between species in the histopathological findings (107). The rhesus monkey is the most susceptible species to viral infection, and therefore a good model for the study of COVID-19, as well as for the development of vaccines and pharmacological studies (107, 108). De Wit et al. (110) studied rhesus monkeys intratracheally inoculated with the virus, finding that viral RNA was detectable in the throat during the first days, but with levels subsequently decreasing until becoming undetectable. Bao et al. (111) and Chandrashekar et al. (112) intratracheally and intranasally inoculated rhesus monkeys with the SARS-CoV-2 virus to study the possibility of reinfection. Williamson et al. (113) used rhesus monkeys that had previously been inoculated through the intranasal, oral, ocular, and intratracheal routes for pharmacological research. These animals have also been used for the development of vaccines (106, 107, 114, 115).

## Models for CNS Research

Coronaviruses have been found in autopsy studies in the CNS of patients with multiple sclerosis, Parkinson’s disease, and Alzheimer disease. Experimental studies have shown that human CoVs can infect neurons, astrocytes, and microglia in primary cultures as well as immortalized human microglial cells (6). The suggestion that SARS-CoV-2 may use the brain as a reservoir (10), potentially favoring the development of neurodegenerative diseases (11), underscores the need to specifically analyze the effect of the virus on the CNS.

## CNS Cell Lines

The information currently available is from research on SARS-CoV, which has 78% nucleotide homology with SARS-CoV-2 (18). Although CNS tropism has been described (20), no specific models of neural cell lines have been developed for the study of SARS-CoV-2; however, neural progenitor cells (NPCs), neurons and microglia derived from human induced pluripotent stem cells (hIPSCs) have already been used in *in vitro* studies of SARS-CoV-2 viral infection, demonstrating the virus potential to infect CNS cells (116–118). Previous studies with other neurotropic human coronaviruses showed possible neural cell lines susceptible to infection by SARS-CoV-2 that may be useful in studying the possible mechanisms by which the virus infects the CNS. Studies into neural susceptibility to SARS-CoV infection have used cell lines including HOG, a line derived from a human oligodendroglioma that expresses proteins characteristic of oligodendrocytes and is very widely used in the study of neurons (119, 120) and the C6 cell line, derived from a glioma induced in Wistar Furth rats exposed to N-nitroso-N-methylurea, which is morphologically similar to glioblastoma multiforme when injected into the brains of neonate rats (121, 122). Although both cell lines have been shown to be susceptible to SARS-CoV infection, low levels of viral replication have been observed in comparison with such other susceptible cell lines as Vero E6 or Caco-2 (123). Other cell lines used to study the virulence of HCoV-229E and HCoV-OC43 in the CNS include human H4 brain neuroglioma cells, the LA-N-5 human neuroblastoma cell line, the CHME-5 human fetal microglia cell line, and the U-373 MG and U-87 MG astrocytic lines derived from a human glioblastoma and an astrocytoma, respectively, among many others (124–129). Cultures of human primary neurons, astrocytes, oligodendrocytes, and microglia have been used to study these viruses (130, 131). All these neural cell lines may be useful in the near future to study SARS-CoV-2.

## Brain Organoids as a Model of CNS Infection by SARS-CoV-2

Organoids are miniaturized, simplified, three-dimensional versions of an organ produced *in vitro*, partially recreating the cellular structure and the functioning of that organ (132, 133). Classic cell culture systems present certain limitations, such as the inability to study complex and dynamic responses or cell-cell interactions. The use of organoids enables us to study complex physiological or pathological processes in structures bearing much greater resemblance to *in vivo* conditions, including SARS-CoV-2 infection, tropisms and potential treatments (133–135). To date, SARS-CoV-2 infection has been studied in human organoids of lung, liver, intestine, blood vessels, and kidney (42, 118, 136–138). Human brain organoids have also been used; these present strong cellular and structural similarities to some mammalian brain regions, such as a neural epithelium containing NPCs, that align to form a ventricular zone-like layer, cortical neurons, that contribute to the formation of a cortical plate-like layer and glial cells, such as astrocytes or oligodendrocytes (139–141). These organoids are useful for the study of early stages of human neurodevelopment and network formation, key cellular processes such as proliferation, differentiation, apoptosis, synaptogenesis or myelination, CNS function such as electrophysiological activity, neurodegenerative diseases, potential treatments, and have been already used for the study of other virus such as ZIKA virus or HIV (139, 141–144).

Ramani et al. (116) observed that in these brain organoids, the virus mainly infects mature cortical neurons and presents a perinuclear distribution within these cells. Furthermore, neurodegenerative effects have been observed in cells infected by SARS-CoV-2, including cell death and hyperphosphorylation, as well as mislocation of Tau protein; these alterations are observed in such conditions as tauopathies or Alzheimer disease (116). However, no productive replication of the virus was observed in these cells, at least in the first 4 days after infection (116), which would support the hypothesis that the CNS may act as a long-term reservoir of the virus (10). In contrast, Bullen et al. (139) observed an incremented accumulation of viral particles in neural cells of brain organoids between 6 and 72 h after SARS-CoV-2 infection, suggesting an active replication and productive infection of the virus in neural cells during the first days. Viral particles were detected mainly in the neuronal soma and, in some cells, also into the neurites (139). Similar to Ramani et al., Mesci et al. (141) used these brain organoids and observed that the virus was able to infect neurons, including NPCs and mature cortical neurons, and cause cell death accompanied by the impairment of excitatory synapses. Furthermore, this work tested the efficiency of Sofosbuvir, an FDA-approved brain-penetrant antiviral drug for positive-sense single-stranded RNA viruses (145), as a treatment for the SARS-CoV-2 infection and observed that this drug was able to rescue the altered synaptogenesis and decrease neuronal death and viral accumulation in these brain organoids (141). Song et al. (117) also demonstrated that SARS-CoV-2 has neuroinvasive capacity in human brain organoids, particularly of NPCs and mature cortical neurons. Infected cells showed a hypermetabolic state and viral particles were accumulated within endoplasmic reticulum-like structures, indicating the virus ability to use the neural cell machinery to replicate (117). In addition, a hypoxic environment and extensive neuronal cell death were observed in high density SARS-CoV-2 infected areas, suggesting that virus infection could promote death of nearby cells (117). Finally, this study detected IgG antibodies against SARS-CoV-2 in the cerebrospinal fluid of a COVID-19 patient that were able to block SARS-CoV-2 infection in brain organoids (117). All these studies show that SARS-CoV-2 can directly infect neural cells and trigger damaging consequences that could cause neurologic symptoms. Also, these studies expose the great potential of the human brain organoids for the study of the SARS-CoV-2 effects in the CNS.

## MHV-CoV Infection in Mice

Mouse hepatitis virus (MHV) is a βCoV that poses no risk to humans but presents a great similarity with other viruses from the same family, such as SARS-CoV, MERS-CoV, and SARS-CoV-2. It penetrates the CNS, causing white matter lesions; it has therefore been proposed as a viral model of demyelinating disease (146, 147). The virus has been shown to remain in the white matter and to be able to replicate in the CNS (148); therefore, it is a good model for the study of CNS infection by coronaviruses. Neurotropic strains of MHV-CoV have been used extensively to induce acute and chronic demyelinating disease mediated by neuroinflammation (149). Depending on the inoculation route and the MHV-CoV strain, different CNS regions are affected. Inoculation with experimental neurotropic strains, especially MHV-A59, induces a biphasic disease of acute meningoencephalitis at 10–14 days after inoculation, followed by a disease causing subacute, chronic inflammatory demyelination in the brain; spinal cord involvement is more pronounced (150). Virus translocation from the initial site of inoculation in the brain to the spinal cord is caused by the transit of virus particles in neural and glial cells, as well as mechanisms that involve the fusion of lipid membranes, probably during the virus internalization step (151). Intranasal and intracranial inoculation of JHM-CoV induces similar symptoms in BALB/c mice to those caused by MHV-A59. After intranasal inoculation of mice, MHV-CoV accesses the CNS through the olfactory nerve and propagates from the olfactory system to limbic system structures and their connections with the brainstem (152).

In order to study the immune system role in demyelination induction caused by MHV infection, Wang et al. (153) treated infected animals with gamma radiation to cause immunosuppression and, subsequently, reconstituted immunity by transferring cells from other immunocompetent animals. The results showed that demyelination was prevented by radiation and was present again when the immunity was restored, indicating that immunity is directly involved in the demyelination process (153). Moreover, CD4 and CD8 T cells have been observed to play a critical role in the development of the demyelinating process, with γδ T cells being the most important for this process (154, 155). In contrast, B cells, the most abundant cell type in the spleen, and NK cells are not involved in demyelination as nude animals without spleen do not present demyelination (154), MHV offers a unique model for studying host defense-mediated demyelination during chronic infection in a phase acute viral infection and immune response (156).

## Conclusion

Research on SARS-CoV-2 has become a necessity due to the magnitude of its spread worldwide. Such aspects as infection rate and mechanisms, the possibility of reinfection, and possible therapeutic approaches make it necessary not only to use the experimental models previously employed to study the SARS-CoV and MERS-CoV coronaviruses, but also to generate new models to respond to urgent questions. The potential involvement of the CNS due to SARS-CoV-2 infection should be studied specifically, and research efforts must focus on obtaining information with cellular or animal models to expand our understanding of the virus.

## Author Contributions

JM-G and UG-P: lead researchers. All authors: research project group, manuscript drafting, and critical review of the manuscript.

## Conflict of Interest

The authors declare that the research was conducted in the absence of any commercial or financial relationships that could be construed as a potential conflict of interest.
