# Supplementary material for: Experimental Models for the Study of Central Nervous System Infection by SARS-CoV-2
Source: Front Immunol. 2020 Aug 28;11:2163. doi: 10.3389/fimmu.2020.02163 (PMC7485091; doi:10.3389/fimmu.2020.02163)
Supplement: Supplementary file 1 [file Data_Sheet_1.PDF]

Supplementary Material 1

Main culture media used in *in vitro* experiments in the SARS-CoV-2 study

Reference	Cell Line	Culture Media
**Caly et al., 2020 (44)**	Vero E6	EMEM with 7% FBS, 2 mM L-Glutamine, 1 mM sodium pyruvate, 1500 mg/L sodium bicarbonate, 15 mM HEPES and 0.4 mg/ml geneticin
**Colson et al., 2020 (43)**	Vero E6	MEM with 4% FCS and 1% glutamine
**Hoffman et al., 2020 (23)**	Vero E6	DMEM with 10% FBS, 100 U/mL of penicillin, 0.1 mg/mL of streptomycin, 1x non-essential amino acid solution and 10 mM sodium pyruvate
**Runfeng et al., 2020 (48)**	Vero E6	DMEM with 10% FBS
**Yao et al., 2020 (47)**	Vero E6	DMEM with 5% FBS
**Hoffman et al., 2020 (23)**	HEK 293T	DMEM with 10% FBS, 100 U/mL of penicillin, 0.1 mg/mL of streptomycin, 1x non-essential amino acid solution and 10 mM sodium pyruvate
**Ou et al., 2020 (39)**	HEK 293T	DMEM with 10% FBS, 100 U/ml penicillin, 100 µg streptomycin and 0.25 µg/ml Fungizone
**Wang et al., 2020 (40)**	HEK 293T	DMEM or RPMI 1640 with 10% FBS, 1% penicillin/streptomycin and 2% L-glutamine
**Hoffman et al., 2020 (23)**	BHK-21	DMEM with 10% FBS, 100 U/mL of penicillin, 0.1 mg/mL of streptomycin, 1x non-essential amino acid solution and 10 mM sodium pyruvate
**Ou et al., 2020 (39)**	BHK-21	DMEM with 10% FBS, 100 U/ml penicillin, 100 µg streptomycin and 0.25 µg/ml Fungizone
**Hoffman et al., 2020 (23)**	Huh-7	DMEM with 10% FBS, 100 U/mL of penicillin, 0.1 mg/mL of streptomycin, 1x non-essential amino acid solution and 10 mM sodium pyruvate
**Runfeng et al., 2020 (48)**	Huh-7	DMEM with 10% FBS
**Wang et al., 2020 (26)**	Huh-7	DMEM with 10% FBS
**Xia et al., 2020 (26)**	Huh-7	DMEM with 100 U/ml penicillin, 100 mg/ml streptomycin and 10% FCS
**Colson et al., 2020 (43)**	LLC-MK2	MEM with 4% FCS and 1% glutamine
**Ou et al., 2020 (39)**	LLC-MK2	DMEM with 10% FBS, 100 U/ml penicillin, 100 µg streptomycin and 0.25 µg/ml Fungizone
**Hoffman et al., 2020 (23)**	Caco-2	MEM with 10% FBS, 100 U/mL of penicillin, 0.1 mg/mL of streptomycin, 1x non-essential amino acid solution and 10 mM sodium pyruvate
**Hoffman et al., 2020 (23)**	Calu-3	MEM with 10% FBS, 100 U/mL of penicillin, 0.1 mg/mL of streptomycin, 1x non-essential amino acid solution and 10 mM sodium pyruvate
**Sheahan et al., 2020 (73)**	Calu-3	DMEM with 20% fetal bovine serum and 1x antibiotic/antimycotic
**Ou et al., 2020 (39)**	Calu-3	DMEM with 10% FBS, 100 U/ml penicillin, 100 µg streptomycin and 0.25 µg/ml Fungizone
**Bullen et al., (141)**	Human brain organoids	Neurobasal electro medium with B27-electro, glutamax, 10ng/mLGDNF and 10ng/mLBDNF
**Mesci et al., 2020 (143)**	Human brain organoids	Neurobasal with GlutaMAX, 1% Gem21 NeuroPlex, 1% NEAA and 1% penicillin/streptomycin
**Ramani et al., 2020 (118)**	Human brain organoids	DMEM/F12 and Neural Basal Medium (1:1 ratio) with 1:200 N2, 1:100 L-glutamine, 1:100 B27 w/o vitamin A, 100 U/mL penicillin, 100 µg/mL streptomycin, 23 µM insulin, 0.05 mM MEM non-essential amino acids, and 0.05 mM β-mercaptoethanol

MEM = Minimum Essential Medium, DMEM = Dulbecco's MEM, EMEM = Earle's MEM, RPMI = Roswell Park Memorial Institute medium, FBS = Fetal Bovine Serum, FCS = Fetal Calf Serum
